# Quantitative myelin water imaging using short TR adiabatic inversion recovery prepared echo-planar imaging (STAIR-EPI) sequence

**DOI:** 10.3389/fradi.2023.1263491

**Published:** 2023-09-28

**Authors:** Hamidreza Shaterian Mohammadi, Dina Moazamian, Jiyo S. Athertya, Soo Hyun Shin, James Lo, Arya Suprana, Bhavsimran S. Malhi, Yajun Ma

**Affiliations:** Department of Radiology, University of California San Diego, San Diego, CA, United States

**Keywords:** myelin water imaging, STAIR, EPI, multiple sclerosis, aMWF

## Abstract

**Introduction:**

Numerous techniques for myelin water imaging (MWI) have been devised to specifically assess alterations in myelin. The biomarker employed to measure changes in myelin content is known as the myelin water fraction (MWF). The short TR adiabatic inversion recovery (STAIR) sequence has recently been identified as a highly effective method for calculating MWF. The purpose of this study is to develop a new clinical transitional myelin water imaging (MWI) technique that combines STAIR preparation and echo-planar imaging (EPI) (STAIR-EPI) sequence for data acquisition.

**Methods:**

Myelin water (MW) in the brain has shorter *T*_1_ and *T*_2_ relaxation times than intracellular and extracellular water. In the proposed STAIR-EPI sequence, a short TR (e.g., ≤300 ms) together with an optimized inversion time enable robust long *T*_1_ water suppression with a wide range of *T*_1_ values [i.e., (600, 2,000) ms]. The EPI allows fast data acquisition of the remaining MW signals. Seven healthy volunteers and seven patients with multiple sclerosis (MS) were recruited and scanned in this study. The apparent myelin water fraction (aMWF), defined as the signal ratio of MW to total water, was measured in the lesions and normal-appearing white matter (NAWM) in MS patients and compared with those measured in the normal white matter (NWM) in healthy volunteers.

**Results:**

As seen in the STAIR-EPI images acquired from MS patients, the MS lesions show lower signal intensities than NAWM do. The aMWF measurements for both MS lesions (3.6 ± 1.3%) and NAWM (8.6 ± 1.2%) in MS patients are significantly lower than NWM (10 ± 1.3%) in healthy volunteers (*P* < 0.001).

**Discussion:**

The proposed STAIR-EPI technique, which can be implemented in MRI scanners from all vendors, is able to detect myelin loss in both MS lesions and NAWM in MS patients.

## 1. Introduction

Myelin is a lipid-protein bilayer that surrounds the axonal fibers of neurons (1). It plays an essential role in normal brain function by facilitating the rapid conduction of action potentials in the axon (2). Many neurological and degenerative diseases, such as multiple sclerosis (MS), are characterized by myelin damage and loss (3, 4). Thus, evaluations of demyelination and remyelination are essential for the accurate diagnosis and treatment monitoring of these diseases. Conventional magnetic resonance imaging (MRI) techniques, such as *T*_1_- and *T*_2_-weighted fast spin echo (*T*_1_w- and *T*_2_w-FSE), provide high soft-tissue contrast and are routinely used in the diagnosis of MS and many other neurological diseases. However, these clinical sequences are unable to distinguish demyelination lesions, such as those observed in MS, from the ones caused by inflammation, edema, axonal loss, or gliosis (5–7). Moreover, it is also difficult for clinical MRI to detect myelin damage in brains that appear to be normal (i.e., where there are no apparent lesions) (8). Consequently, developing myelin-specific imaging techniques is of critical importance to improved evaluation of neurological diseases in clinical practice.

In the last two decades, many myelin water imaging (MWI) techniques have been developed for the specific evaluation of myelin changes. Myelin water (MW) is the water component tightly bound or trapped in the myelin bilayer (9–14). It has much shorter *T*_1_ and *T*_2_ relaxation times than intracellular/extracellular water. Myelin water fraction (MWF), defined as the signal ratio of MW to total water, is the biomarker used to quantify MW content changes (15–18).

State-of-the-art MWI techniques like multi-echo spin echo *T*_2_ relaxometry (15), multicompartment analysis of *T*_2_* decay (16), and multicomponent-driven equilibrium single-pulse observation of *T*_1_ and *T*_2_ (mcDESPOT) (17) have been developed to quantify MWF and have been applied for the assessment of MS. The high correlation between the MRI-measured MWF and histologically quantified myelin content has been demonstrated in brain sample studies (19, 20). Moreover, all these techniques are able to detect significant MWF decreases in demyelinated lesions in patients with MS in comparison to the normal white matter (NMW) in healthy individuals (15–17, 21, 22). Despite the success of these myelin-specific techniques, studies have found that these multicompartment modeling techniques are sensitive to system flaws such as *B*_1_ and *B*_0_ inhomogeneities (23–27). Different data post-processing strategies may also produce different results (28–31). Consequently, these techniques suffer from limited accuracy and robustness in terms of MWF estimation, shortcomings that slow down their clinical translation.

Another promising technique that has been developed for selective imaging of MW, known as direct visualization of short transverse relaxation time component (ViSTa), is based on the *T*_1_ difference between MW and long *T*_2_ intracellular/extracellular water components (32). The *T*_1_ relaxation times for the long *T*_2_ components in white matter and gray matter range from 750 to 1,000 ms and from 1,300 to 1,800 ms, respectively, at 3T (32–34). In contrast, the *T*_1_ relaxation times for MW components are typically shorter than 600 ms (17, 18, 35, 36). This technique employs a double inversion recovery (DIR) preparation to robustly suppress all the long *T*_1_ water components in the brain. Moreover, not only is ViSTa less sensitive to *B*_1_ and *B*_0_ inhomogeneities, but it does not require any complicated modeling to quantify MWF. That being said, ViSTa's scan time is too long for clinical use for whole brain coverage (∼3 min per slice).

Most recently, Ma et al. have developed a short TR adiabatic inversion recovery (STAIR) technique in combination with a 3D Cones acquisition (STAIR-Cones) for time-efficient selective MWI in the whole brain (37). This technique uses a short TR to suppress long *T*_1_ water components with a variety of *T*_1_s. Similar to ViSTa, the STAIR sequence is relatively insensitive to *B*_1_ and *B*_0_ inhomogeneities because it uses an adiabatic full passage (AFP) pulse for signal inversion; however, because the 3D Cones sequence is not a clinically available sequence (it is primarily available in GE research scanners), the STAIR-Cones sequence is currently limited in its potential for clinical translation.

In this study, we proposed a new clinically translational MWI sequence for whole-brain MWI and quantification, which is a combination of the STAIR technique and echo-planar imaging (EPI) readout. Unlike the research-dedicated Cones sequence, the EPI sequence is a routinely used clinical sequence that is available to all vendors, posing the proposed STAIR-EPI as a technique with greater potential in clinical translation than STAIR-Cones. Moreover, similar to the ViSTa and STAIR-Cones techniques, the proposed STAIR-EPI does not require complicated post-processing such as solving the ill-conditioned problems for those multicompartment modeling techniques (15–17). We investigated the feasibility of the proposed STAIR-EPI technique for the quantification of MWF and compared its values in the lesions and normal-appearing white matter (NAWM) of seven MS patients against the values of NWM of seven healthy volunteers on a clinical 3 T MRI scanner.

## 2. Materials and methods

### 2.1. STAIR-EPI sequence

**Figure 1:**
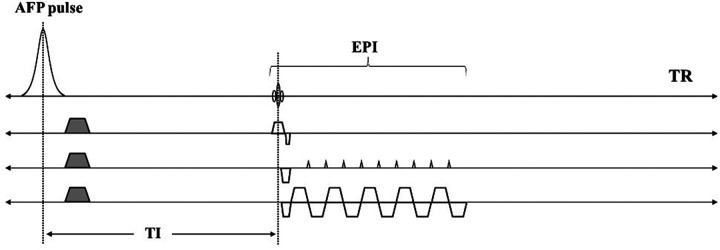
Sequence diagrams for the STAIR-EPI sequence. An AFP pulse and a short TR (e.g., 250 ms) together with an optimized TI in STAIR-EPI enables robust long *T*_1_ water suppression. The EPI allows fast data acquisition of the remaining MW signals.

Figure 1 shows a diagram of the STAIR-EPI sequence. After an AFP pulse and duration of longitudinal magnetization recovery, a blipped multi-shot EPI is used for fast data acquisition. The EPI starts with a 90° radiofrequency (RF) excitation pulse. Inversion time (TI) is determined as the time interval between the center of AFP to the center of the excitation pulse. A short TR between 180 and 300 ms is typically used in the STAIR sequence and, with an appropriate TI (37), signals from long *T*_1_ water components with a broad range of *T*_1_s (in this case, 600–2,000 ms) can be well suppressed.

The signal equation for the STAIR-EPI sequence is expressed as follows:


(1) $$S_{STAIR}=M_{0}(1-Qe^{-TR/T_{1}}-(1-Q)e^{-TI/T_{1}})e^{-\frac{TE}{T_{2}^{*}}}.$$

$M_{0}=[M_{0}^{\mathrm{MW}},\;M_{0}^{L}]$ are the longitudinal magnetizations of MW and long *T*_1_ water components in the equilibrium state. $S_{\mathrm{STAIR}}=[S_{\mathrm{STAIR}}^{\mathrm{MW}},\;S_{\mathrm{STAIR}}^{L}]$ are the signal intensities of MW and long *T*_1_ water components in STAIR-EPI imaging. Q represents the inversion efficiency for the AFP pulse with a range of −1 (full inversion) to 1 (no disturbance to the z-magnetization). For long *T*_1_ water components, Q is assumed to be −1. However, for MW with a short *T*_2_* of 10 ms, Q is set to −0.75 (i.e., $Q_{\mathrm{MW}}=0.75$) when a relatively long AFP pulse (i.e., 8.64 ms) is utilized for signal inversion based on Bloch simulation (37–40). Our previous numerical simulation demonstrated that a TR range of [180, 300] ms provides a reasonable contrast-to-noise ratio (CNR) efficiency in STAIR MWI (37). Similar to the STAIR-Cones for MWI, a TR of 250 ms was selected for STAIR-EPI imaging in this study. The corresponding optimal TI (i.e., $TI_{\mathrm{optimal}}$) of the STAIR-EPI sequence is determined by minimizing the signals of long *T*_1_ components with a wide range of *T*_1_ values [i.e., (600, 2,000)ms] [see Equation (11) in Ref. (37)]. With the determined $TI_{\mathrm{optimal}}$, the MW signal can be expressed as follows:


(2) $$S_{STAIR}^{MW}\;=\;M_{0}^{MW}(1\;-\;Q_{MW}e^{-TR/T_{1}}\;-\;(1\;-\;Q_{MW})e^{-TI_{optimal}/T_{1,MW}})e^{-\frac{TE}{T_{2,MW}^{*}}}.$$

$T_{1,\mathrm{MW}}$ and $T_{2,\mathrm{MW}}^{*}$ are $T_{1}$ and $T_{2}^{*}$ of MW respectively. To facilitate the quantification of apparent MWF (aMWF), a proton density-weighted EPI (PD-EPI) sequence is also scanned for total water imaging. The signal equation of the PD-EPI sequence is expressed as follows:


(3) $$S_{PD}=M_{0}^{total}e^{-\frac{TE}{T_{2,total}^{*}}}.$$

$M_{0,\mathrm{total}}$ and $T_{2,\mathrm{total}}^{*}$ are the equilibrium longitudinal magnetization and $T_{2}^{*}$ of total water respectively.

aMWF is defined as the PD ratio of MW to total water, and is expressed as follows:

(4) $$aMWF=\frac{M_{0}^{MW}}{M_{0}^{total}}.$$

With known signal intensities of MW and total water (i.e., $S_{\mathrm{STAIR}}^{\mathrm{MW}}$ and $S_{\mathrm{PD}}$), the aMWF can be easily computed by the division operation between Equations (2) and (3). The *T*_1_ and *T*_2_* values of MW ($T_{1,\mathrm{MW}}$ and $T_{2,\mathrm{MW}}^{*}$) are set to 220 and 10 ms respectively (16–18, 23, 35, 36). The *T*_2_* of total water ($T_{2,\mathrm{total}}^{*}$) is set to 60 ms (16, 23).

### 2.2. In vivo study

This study was approved by our institutional review board (IRB) and informed consent was obtained from all participants. Seven healthy volunteers (mean age: 39.9 ± 15.9 years, 3 males and 4 females) and seven MS patients (mean age: 53.7 ± 8.7 years, 2 males and 5 females) were recruited and underwent MRI scans. The inclusion criteria for the disease group included a documented diagnosis of MS and age over 18; exclusion criteria included concomitant malignancy and other severe diseases like stroke. The inclusion criteria for healthy volunteers were that participants were in good health and over 18 years old. All individuals with any contraindications for MRI were excluded from study participation.

All participants were scanned on a 3 T clinical MRI scanner (MR750, GE Healthcare Technologies, Milwaukee, WI) and a 12-channel head coil was employed for signal reception.

The sequence parameters of the STAIR-EPI and PD-EPI sequences were as follows: i) STAIR-EPI: field of view (FOV) = 22 × 22 cm^2^, matrix = 128 × 128, TR/TI/TE = 250/117/5.5 ms, flip angle (FA) = 90°, number of shots = 8, slice thickness = 5 mm, number of slices = 15, number of excitations (NEX) = 30, and scan time = 15 min; (ii) PD-EPI: FOV = 22 × 22 cm^2^, matrix = 128 × 128, TR/TE = 250/5.5 ms, FA = 5°, slice thickness = 5 mm, number of slices = 15, NEX = 10, and scan time = 43s. A clinical *T*_2_-FLAIR sequence included for diagnosis was scanned with the following parameters: FOV = 25.6 × 25.6 × 16.3 cm^3^, matrix = 256 × 256 × 136, TR/TI/TE = 7,000/2,028/130 ms, acceleration factor = 4, and scan time = 5.5 min.

### 2.3. Data analysis

**Figure 2:**
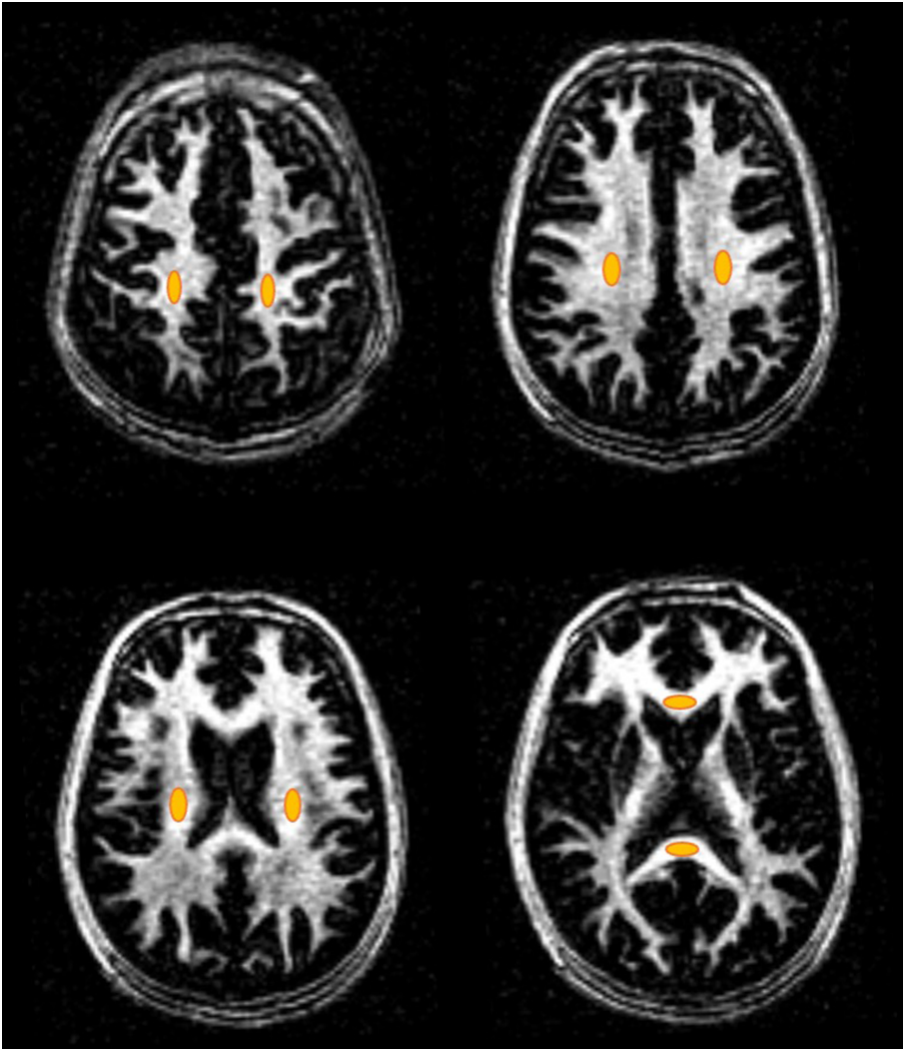
Representative non-lesion ROIs (orange ovals) of the eight WM regions including the left and right centrum semioval, subcortical white matter, periventricular regions, splenium, and genu of the corpus callosum for healthy volunteers and MS patients.

MS lesion regions in MS patients (a total of 66 lesions) and eight non-lesion white matter regions in healthy volunteers (i.e., NWM) and MS patients (i.e., NAWM) were manually drawn for aMWF quantification. The non-lesion regions included the left and right centrum semioval, subcortical white matter, periventricular regions, splenium, and genu of the corpus callosum (see Figure 2). Region of interest (ROI) drawings and aMWF calculations were both performed on MATLAB 2022a software (MathWorks Inc., Natick, MA, USA).

A comparison of aMWF measurement was made between NWM in healthy volunteers, NAWM in MS patients, and MS lesions in MS patients. Upon confirming normal distribution through the Kolmogorov-Smirnov test, a one-way ANOVA test was carried out to assess the differences in aMWF among these three groups (i.e., NWM, NAWM, and MS lesions). A *post hoc* test (Games-Howell test) was conducted for paired comparisons between each of the two groups (i.e., NWM vs. NAWM, NWM vs. MS lesions, and NAWM vs. MS lesions). *P* values less than 0.05 indicates statistical significance.

## 3. Results

**Figure 3:**
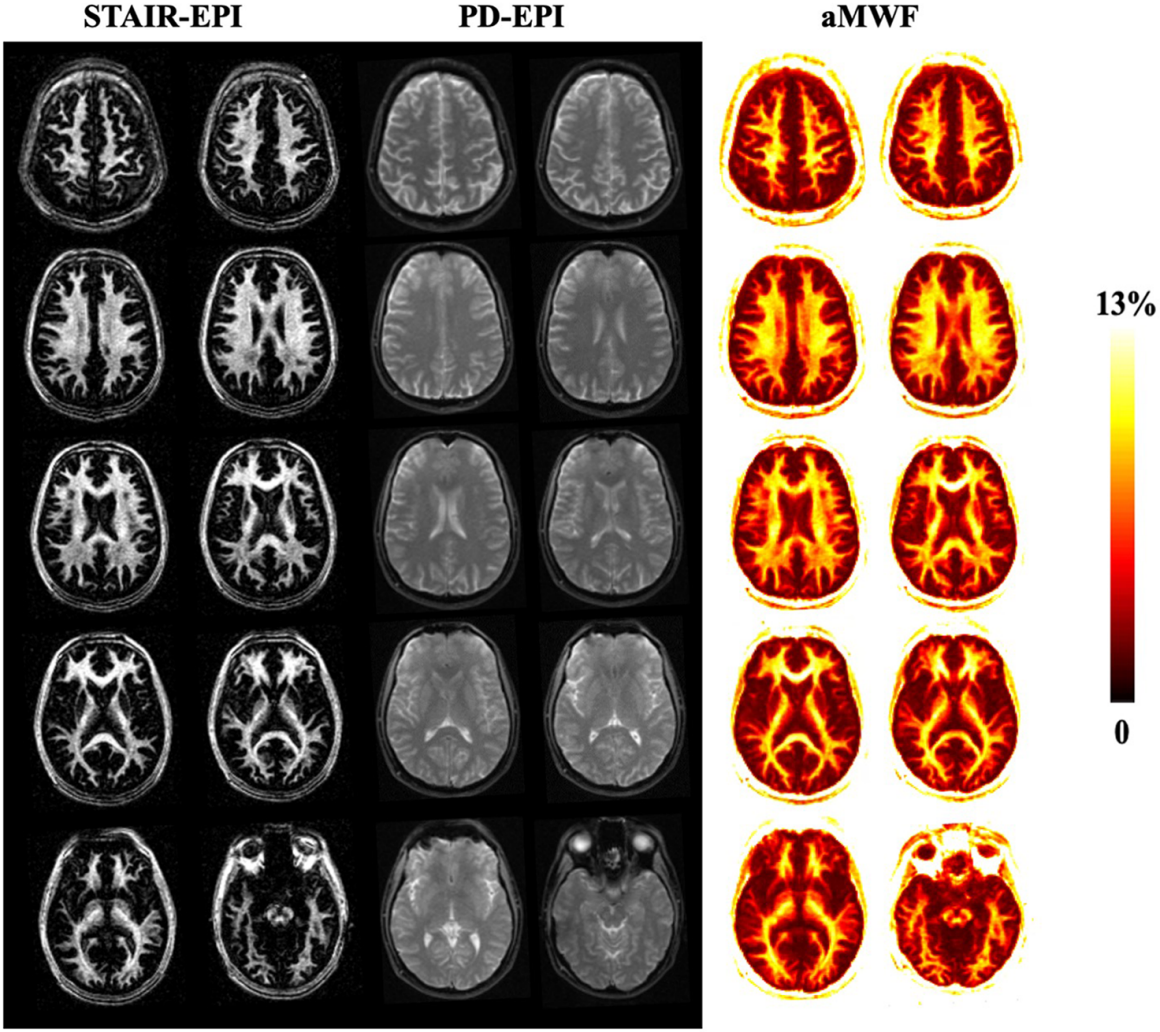
Representative STAIR-EPI (columns 1 and 2) and PD-EPI (columns 3 and 4) images, as well as the corresponding aMWF maps (columns 5 and 6) from a 31-year-old healthy male volunteer. White matter regions have a much higher myelin water content than gray matter regions.

Figure 3 shows the representative STAIR-EPI and PD-EPI images as well as corresponding aMWF maps from a 31-year-old male healthy volunteer. Much higher MW signal intensities in the STAIR-EPI images are found in white matter regions than those in grey matter regions. The aMWF maps also demonstrate a higher aMWF in the white matter region than in the grey matter region.

**Figure 4:**
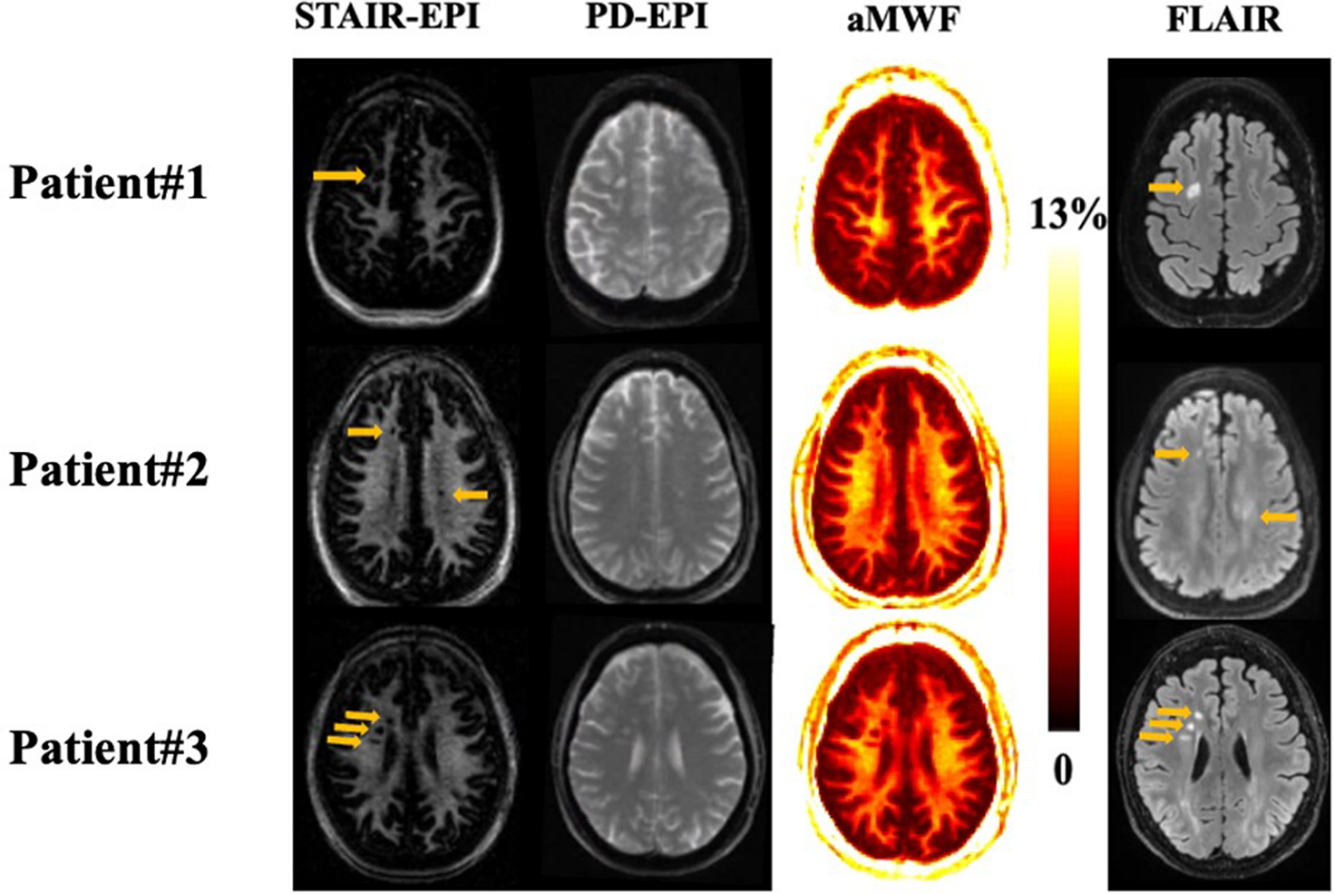
Representative STAIR-EPI (first column), PD-EPI (second column), and aMWF (third column), as well as *T*_2_-FLAIR (fourth column) from three patients with MS (patient #1 is a 57-year-old female, patient #2 is a 52-year-old male, and patient #3 is a 67-year-old female). Hyperintense lesions detected on *T*_2_-FLAIR images (arrows) show a signal loss on the corresponding myelin water images and aMWF maps.

Figure 4 shows representative *T*_2_-FLAIR, PD-EPI, and STAIR-EPI images as well as aMWF maps from three MS patients. The hyperintense lesions in *T*_2_-FLAIR show low signal intensities in the STAIR-EPI images and also have lower aMWF values than NAWM regions, demonstrating a decrease in MW content for these MS lesions.

Table 1 summarizes the mean and standard deviation (SD) values of the aMWF measurements from all participants. The aMWF value of NWM in healthy volunteers is 10 ± 1.3%, while the aMWF values of NAWM and MS lesions in MS patients are 8.6 ± 1.2% and 3.6 ± 1.3%, respectively. Significant difference is achieved between these three groups (*p* < 0.001).

**Table 1:** Mean and standard deviation (SD) of aMWF measurements from NWM, NAWM, and MS lesions as well as one-way ANOVA test results for these three groups.

	aMWF	95% CI	*P* Value
	(%, mean ± STD)		(one-way ANOVA test)
NWM	9.9 ± 1.3%	9.6–10.3	<0.001
NAWM	8.5 ± 1.2%	8.2–8.8	
MS Lesion	3.6 ± 1.3%	3.3–3.9	

Significant difference is achieved between the three groups (*p* < 0.001). Confidence intervals (CI) at a 95% confidence level provide US with a range of values that is likely to include the true population mean for each group.

**Figure 5:**
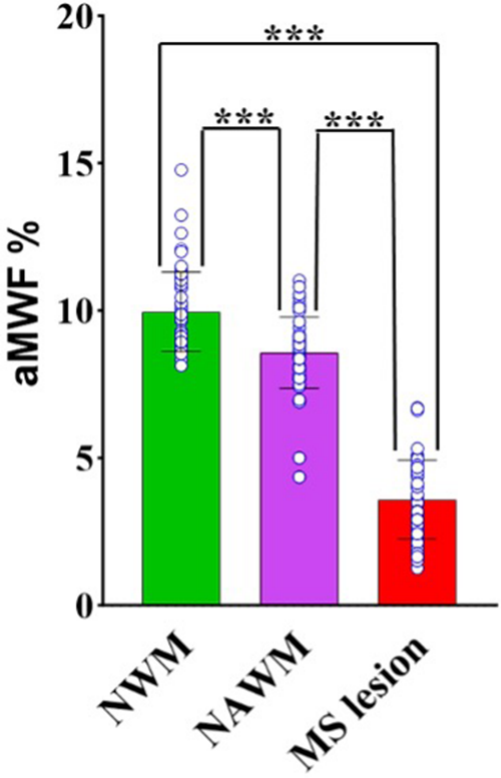
Paired comparisons of aMWF measurements between NWM, NAWM, and MS lesions. The individual data points for each group are also shown in the bar plots. Significantly lower aMWF values are found in both MS lesions and NAWM regions in MS patients in comparison with NWM in healthy volunteers. (“***” indicates *p* < 0.001).

Figure 5 shows the paired comparison of measured aMWF values on each of the two groups (i.e., NWM vs. NAWM, NWM vs.MS lesions, and NAWM vs. MS lesions). The results indicate a notable distinction of aMWF measurements between MS lesions against both NAWM and NWM (*p* < 0.001). A significant difference in aMWF measurement is also observed between NAWM and NWM (*p* < 0.001). These results demonstrate the feasibility of the STAIR-EPI technique in the detection of demyelination in MS.

## 4. Discussion

In this study, we developed a new translational STAIR-EPI sequence for selective MWI and aMWF quantification in the whole brain. In MS patients, the hyperintense MS lesions in *T*_2_-FLAIR showed low signal intensities in the STAIR-EPI images, indicating high sensitivity in the detection of demyelination for the STAIR-EPI technique. Moreover, the measured aMWF values of MS lesions (3.6 ± 1.3%) and NAWM (8.6 ± 1.2%) in MS patients were significantly lower than those of NWM (10 ± 1.3%) in healthy volunteers. This study reveals the effectiveness of the STAIR-EPI technique in detecting myelin loss in both MS lesions and NAWM in MS patients, a capability that could subsequently be applied in clinical MRI scanners from all vendors. The implementation of the proposed STAIR-EPI sequence is relatively easy and requires neither specialized involvement from scanner vendors nor from application specialists.

EPI is one of the fastest MRI sequences and has already made significant contributions to clinical diagnosis and scientific investigation for various parts of the body including the brain, abdomen, and pelvis (41). With single-shot EPI, a complete set of spatial-encoding data can be gathered following a single RF excitation. However, single-shot EPI suffers from strong imaging distortion induced by eddy currents and *B*_0_ inhomogeneity due to the low bandwidth in the phase-encoding direction. Multi-shot EPI strategy mitigates the imaging distortion because of its increased bandwidth in the phase-encoding direction. In this study, the STAIR technique was combined with the multi-shot EPI acquisition scheme to achieve relatively high-quality MWI. The image quality improved with more shots, but more shots also led to increased scan time (42). We found that eight shots provided a reasonable level of image quality within an acceptable 15-minute scan time.

One of the major advantages of the STAIR-EPI technique is its simplicity, given that it requires neither complex sequence implementation nor complicated post-processing in comparison to conventional techniques (24, 28–30, 37). Additionally, the STAIR preparation is relatively insensitive to *B*_0_ and *B*_1_ field inhomogeneities because it uses the AFP pulse for magnetization inversion (43). These benefits make the STAIR-EPI technique well-suited for clinical practice. Both DIR and STAIR are effective techniques for selective imaging of short *T*_1_ MW and signal suppression of long *T*_1_ intracellular and extracellular water in the brain (32, 37). However, the STAIR technique has an improved scan efficiency over the DIR technique because of its much shorter TR.

Previous research on the MWF quantification in NWM has presented a range of values using different methodologies (15–18, 23, 26, 44–49). For example, multicomponent *T*_2_ decay analysis estimated MWFs ranging from 9% to 15.6% (15, 44–48), whereas multicomponent *T*_2_* decay analysis yielded an MWF range of 6.9% to 14.4% (9, 16, 23, 26, 49). Labadie et al. employed multicomponent *T*_1_ modeling and determined an MWF of 8.3% (18). Ma et al., employing the 3D STAIR-Cones technique, found an MWF value of 9.2% (37). Moreover, a range of MWF values has also been reported for MS lesions in previous MWI studies (15, 37, 48, 49). The multicomponent *T*_2_ analysis indicated MWFs ranging from 1.7% to 6.4% (15, 48), while a multicomponent *T*_2_* analysis determined an MWF of ∼0% (49). For the most recent STAIR-Cones study, Ma et al. reported an MWF value of 4.5% for MS lesions (37). In this study, the mean MWF values of NWM (i.e., 10 ± 1.3%) and MS lesions (i.e., 3.6 ± 1.3%) are consistent with those reported in previous research.

As reported in previous studies, the multicomponent T_2_ relaxometry sequence took between 25 and 38 min to complete (45, 46), whereas the multicomponent *T*_2_* relaxometry sequence took between 20 and 30 min (16, 26). In comparison, the total scan time for the proposed STAIR-EPI technique is around 16 min, which is relatively shorter than the typical multicomponent *T*_2_ and *T*_2_* relaxometry techniques.

There were some limitations in this study. First, only seven MS patients were scanned in this technical feasibility study. We plan to recruit more patients to investigate demyelination or remyelination in the future. Second, as proof of concept, a relatively large NEX (i.e., 30) was used in the STAIR-EPI scan to achieve a high SNR performance, significantly prolonging the scan time for whole brain coverage. The scan time could be reduced by using a lower NEX value (e.g., 10 or less). Moreover, the recent development of the denoising technique via deep learning could significantly increase the image SNR, thereby facilitating sufficient SNR improvement with a much reduced NEX (e.g., 5) (50, 51).

## 5. Conclusion

The STAIR-EPI technique detects demyelination in MS, facilitating easy clinical translation for the whole brain MWI.

## Data Availability

The raw data supporting the conclusions of this article will be made available by the authors, without undue reservation.
